# Efficacy of a Computerized Simulation in Promoting Walking in Individuals With Diabetes

**DOI:** 10.2196/jmir.1965

**Published:** 2012-05-10

**Authors:** Bryan Gibson, Robin L Marcus, Nancy Staggers, Jason Jones, Matthew Samore, Charlene Weir

**Affiliations:** ^1^George E Whalen Salt Lake City Veterans Affairs Healthcare SystemIDEAS centerSalt Lake City, UTUnited States; ^2^Department of Biomedical InformaticsUniversity of UtahSalt Lake City, UTUnited States; ^3^Department of Physical TherapyUniversity of UtahSalt Lake City, UTUnited States; ^4^Department of Nursing InformaticsUniversity of MarylandBaltimore, MDUnited States; ^5^Kaiser Permanente Southern CaliforniaPasadena, CAUnited States

**Keywords:** Computer simulation, type 2 diabetes mellitus, physical activity, blood glucose

## Abstract

**Background:**

Regular walking is a recommended but underused self-management strategy for individuals with type 2 diabetes mellitus (T2DM).

**Objective:**

To test the impact of a simulation-based intervention on the beliefs, intentions, knowledge, and walking behavior of individuals with T2DM. We compared two versions of a brief narrated simulation. The experimental manipulation included two components: the presentation of the expected effect of walking on the glucose curve; and the completion of an action plan for walking over the next week. Primary hypotheses were (1) intervention participants’ walking (minutes/week) would increase more than control participants’ walking, and (2) change in outcome expectancies (beliefs) would be a function of the discrepancy between prior beliefs and those presented in the simulation. Secondary hypotheses were that, overall, behavioral intentions to walk in the coming week and diabetes-related knowledge would increase in both groups.

**Methods:**

Individuals were randomly assigned to condition. Preintervention measures included self-reported physical activity (International Physical Activity Questionnaire [IPAQ] 7-day), theory of planned behavior-related beliefs, and knowledge (Diabetes Knowledge Test). During the narrated simulation we measured individuals’ outcome expectancies regarding the effect of exercise on glucose with a novel drawing task. Postsimulation measures included theory of planned behavior beliefs, knowledge, and qualitative impressions of the narrated simulation. The IPAQ 7-day was readministered by phone 1 week later. We used a linear model that accounted for baseline walking to test the main hypothesis regarding walking. Discrepancy scores were calculated between the presented outcome and individuals’ prior expectations (measured by the drawing task). A linear model with an interaction between intervention status and the discrepancy score was used to test the hypothesis regarding change in outcome expectancy. Pre–post changes in intention and knowledge were tested using paired *t *tests.

**Results:**

Of 65 participants, 33 were in the intervention group and 32 in the control group. We excluded 2 participants from analysis due to being extreme outliers in baseline walking. After adjustment for baseline difference in age and intentions between groups, intervention participants increased walking by 61.0 minutes/week (SE 30.5, *t*_58 = 1.9,_ *P *= .05) more than controls. The proposed interaction between the presented outcome and the individual’s prior beliefs was supported: after adjustment for baseline differences in age and intentions between groups, the coefficient for the interaction was –.25, (SE 0.07, *t*_57 = –3.2,_ *P *< .01). On average participants in both groups improved significantly from baseline in intentions (mean difference 0.66, *t*_62 = 4.5,_ *P *< .001) and knowledge (mean difference 0.38, *t*_62 = 2.4,_ *P *= .02).

**Conclusions:**

This study suggests that a brief, Internet-ready, simulation-based intervention can improve knowledge, beliefs, intentions, and short-term behavior in individuals with T2DM.

## Introduction

Type 2 diabetes mellitus (T2DM) affects approximately 24 million people in the United States, and is associated with significant morbidity and early mortality [1]. Regular physical activity has been shown to improve glycemic control [2,3], reduce blood pressure [4], reduce lipids [4], and improve cardiorespiratory fitness in individuals with T2DM [5]. These intermediate outcomes have been associated with diabetes-related morbidity and mortality [6]. Although physical activity is considered one of the three pillars of diabetes self-management [7], most people with T2DM do not perform sufficient amounts [8].

There are many reasons why individuals with T2DM may not perform an appropriate self-management behavior such as being active. In this study we used a brief, narrated simulation to address two factors that we believe are amenable to an informatics intervention: inaccurate mental models of the effects of behavior on the disease [9-11] and difficulties in translating good intentions into action [12].

### Glucose Curves

The intervention in this study was based on simulated glucose curves. Glucose curves represent an individual’s variation in plasma glucose through a day. Prior work suggests that glucose curves may be useful as an interface for educational and motivational interventions. Small trials of participants with type 1 diabetes have shown that classroom education using simulated glucose curves positively affects knowledge [13], the frequency of hypoglycemic events [14], and hemoglobin A1c [14]. In T2DM, interviews with individuals before and after viewing their own glucose curves suggest that viewing the curves appears to provide individuals with a greater understanding of the daily variation in glucose (particularly postprandial peaks) and may result in greater intention to perform self-care activities, including to be more physically active [15]. We believe glucose curves offer value because they provide contextual information that individual self-monitored glucose values do not provide.

### Theory of Planned Behavior

According to the theory of planned behavior, an individual’s intention to perform a behavior is a function of their beliefs. In this study we focused on a particular type of belief: *o* *utcome expectancies*. Outcome expectancies are an individual’s belief regarding the likely outcome of a given behavior. The intervention version of our simulation demonstrates the expected change in the glucose curve with both a single walk and regular walking over time.

Prior work has shown that outcome expectancies are related to self-care behaviors in individuals with T2DM [16-19] and that individuals with T2DM generally have low outcome expectancies regarding the effect of exercise on blood glucose [19]. We are not aware of studies that have attempted to *change *outcome expectancies in this population. In general, interventions targeted at outcome expectancies related to physical activity have shown limited efficacy in most populations [20].

### Implementation Intentions

While the beliefs included in the theory of planned behavior have been shown to predict the intentions of individuals with T2DM to be physically active [16], changes in behavioral intention are only moderately predictive of actual changes in behavior [21]. *Implementation intentions *are if–then plans linking specific cues in the environment to a desired behavior. Implementation intentions have been found to be strongly effective in translating intentions into action [12,22]. Recent evidence suggests that individuals who mentally simulate the behavior as they create the implementation intention are even more successful in acting on their intentions [23,24].

The intervention version of our simulation guided participants through writing an action plan for walking while concurrently mentally simulating the planned behavior. In this plan participants indicated where, when, with whom, and for how long they would walk for each day in the next week.

Our hypotheses in this trial were that (1) individuals viewing the intervention version of the narrated simulation would report more walking in the subsequent week than control participants would, and (2) changes in outcome expectancies for intervention participants would vary as a function of the discrepancy between the effect presented in the simulation and the individual’s prior beliefs. Finally, we hypothesized that, overall, both groups would increase their behavioral intentions to walk in the subsequent week and their diabetes-related knowledge.

## Methods

### Participants

We recruited participants between March 2010 and August 2011 at the George E. Whalen Department of Veterans Affairs Medical Center (Salt Lake City, UT, USA) in primary care clinics, diabetes education and weight management classes, a biweekly diabetes exercise group at the University of Utah, a community diabetes health fair, and via an email to a diabetes-related listserv.

Our inclusion criteria were that participants be between 30 and 70 years of age, have a diagnosis of T2DM, and be able to speak English fluently. Participants with a diagnosis of dementia or severe mental disease, using insulin, or having microvascular or macrovascular complications of diabetes were excluded. The rationale for these last two criteria was 2-fold: first, the content of the narrated simulation is geared toward individuals taking oral medications, and second, we wanted to minimize the risk of walking-induced hypoglycemia, foot ulceration, or a cardiac event. Initial recruitment efforts were exclusively among veterans at the Salt Lake City Veterans Administration Healthcare System, aged 40–60 years; however, due to slow recruitment, in June 2010 we expanded recruitment to the larger community and a wider age range.

### Settings

The study was conducted in a location convenient to the participant. These locations included the Salt Lake City VA library, a room adjacent to the exercise room at the diabetes exercise group, a table at a diabetes health fair, a meeting room at a public library, and a private office. All meetings were between the principal investigator (BG) and individual participants.

### Description of the Simulation

The narrated simulation is based on simulated glucose curves [25]. Concepts are presented using the curves without numbers, supplemented by simple icons. A voiceover and music soundtrack accompany the narrated simulation (see Multimedia Appendix 1 and Multimedia Appendix 2 for the intervention and control simulations). Table 1 lists the concepts addressed in the narrated simulation and the time used to explain each concept.

**Figure 1:**
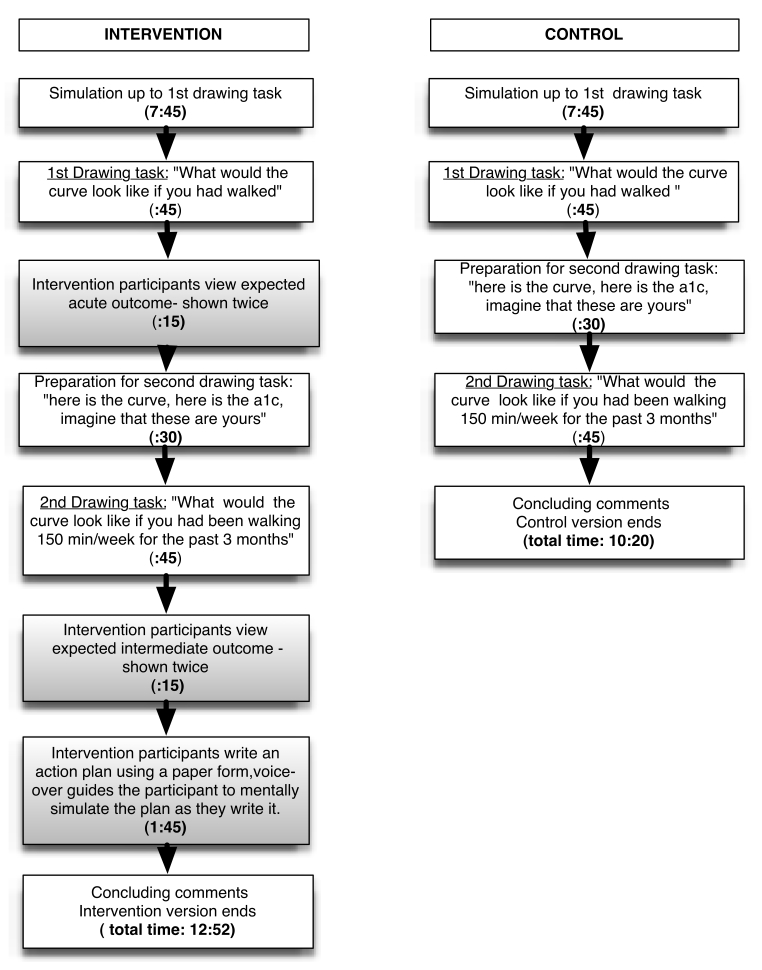
Procedures in the simulation for the intervention and control groups. Boxes with a gray background show intervention-specific components. Duration is in minutes and seconds.

Participants were shown one of two versions of the simulation. The intervention version and the control version were identical through the first 8 minutes and 30 seconds (Figure 1).

**Table 1:** Concepts included in the narrated simulation and their timing.

Concept	Timing (minutes and seconds)
What is the glucose curve?	1:40
When is blood sugar highest and when is it lowest?	0:20
How do meals affect the glucose curve?	0:30
What is the dawn phenomenon?	0:30
What is the safe range of blood sugar?	0:40
What is hemoglobin A1c?	0:15
How does the blood sugar curve change (over years) as A1c increases?	1:40
Why is high blood sugar bad for you? (Includes photographs of individuals with microvascular complications)	1:40
How are changes in A1c associated with complications?	0:20
What can you do today to control your blood sugar?	0:35

### Drawing Tasks

**Figure 2:**
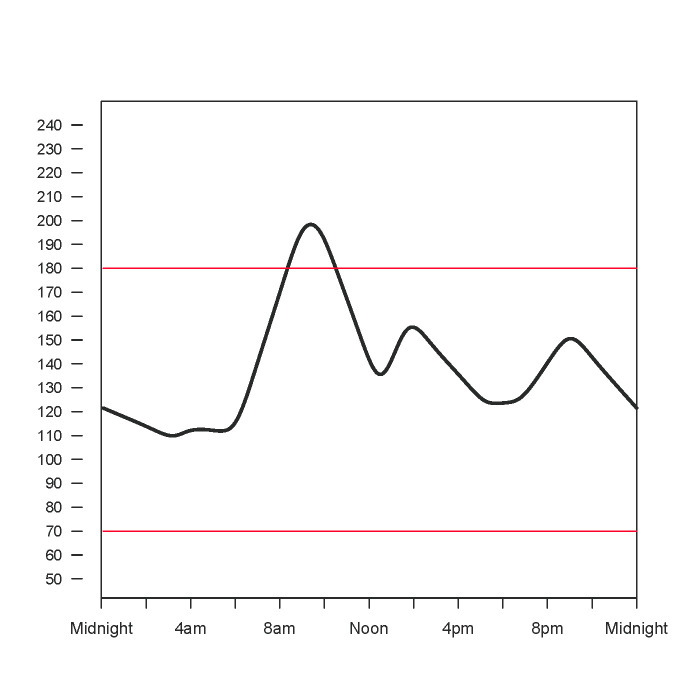
Simulated glucose curve used in the drawing task.

At this point in the narrated simulation, participants were shown a glucose curve of an individual “who has had diabetes for a few years,” and the voiceover asked them to imagine that the curve was their glucose curve from yesterday. Using a paper copy of the curve on the screen (Figure 2), participants were asked to draw what they thought the curve would have looked like if they had gone for a “30-minute walk yesterday an hour after breakfast.” As a second drawing task participants were shown the same curve of an individual “who has had diabetes for a few years,” asked to imagine that it was their curve from yesterday, and asked to draw what they thought the curve would have looked like if they had been walking 5 days a week for 30 minutes each for the past 3 months. The purpose of these two drawing tasks was to capture the individual’s outcome expectancy regarding the change in glucose with a single walk and the change in hemoglobin A1c with regular walking. The advantage of this method is that it allowed us to measure the individuals’ outcome expectancy across three dimensions: the magnitude, direction, and duration of change in the curve.

### Difference Between Control and Intervention Conditions

**Figure 3:**
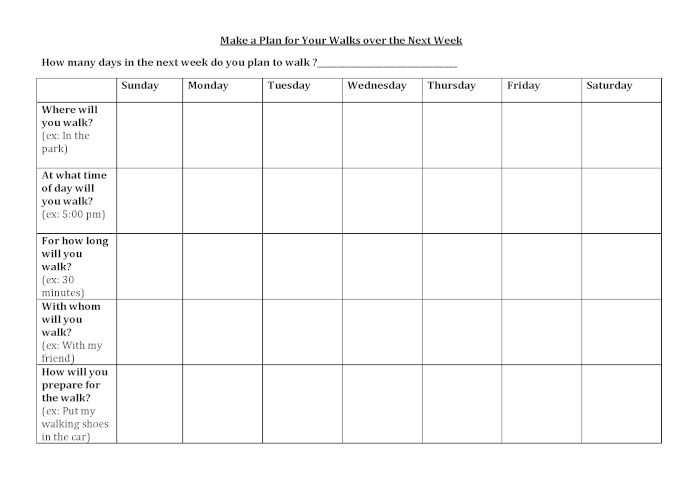
Walking plan to be completed by intervention participants.

The control version of the narrated simulation ended after the two drawing tasks. In the intervention version of the narrated simulation, after completing each drawing task, viewers were shown the expected change in the curve. They were then guided by the voiceover to complete a paper plan of their walking over the next week: how many days they would walk, on which days they would walk, how long each walk would be, in what location they would walk, at what time of day, with whom, and any preparatory actions they would take to facilitate the plan (eg, put walking shoes in their car) (Figure 3). As participants completed the paper plan, the voiceover guided them to mentally simulate the plan. These procedures were specifically designed to facilitate the formation of implementation intentions in the minds of the participants.

### Motivational Components of Both Versions of the Simulation

We hypothesized that two components of the simulation might increase behavioral intentions for both groups. First, in the elicitation of individual’s outcome expectancies via the drawing task, the potential outcome of exercise is framed as an upward counterfactual (how could things have been better: “what *would have *happened if you had exercised”). This was done based on theory [26] and evidence indicating that upward counterfactual thinking facilitates behavioral intentions [27]. Second, the narrated simulation presented the long-term outcomes of being sedentary to both groups (“here is how the glucose curve changes over years if you don’t eat right and exercise regularly”). We included this component based on Williams and colleagues’ suggestion that the construct of outcome expectancy in physical activity research should incorporate both the positive effects of increased activity and the negative effects of being sedentary [20]. We included these components because we wanted to maximize intentions in the intervention group prior to their writing the action plan for walking in the next week; this was based on prior evidence that implementation intentions are most effective when intentions are strong [28]. We did not manipulate these constructs across conditions in this study because our goal was to experimentally determine the effect of the combination of presenting potential outcomes and action planning on behavior.

### Procedures

After obtaining informed consent from the participants, we collected the following measures: (1) demographic information (Table 1), (2) a 10-item version of the Diabetes Numeracy Test [29], (3) a 14-item questionnaire that measures constructs from the theory of planned behavior; this was developed in pilot testing using procedures described by Azjen [30] (see Multimedia Appendix 3 for the complete questionnaire), (4) the short telephone version of the International Physical Activity Questionnaire (IPAQ), a validated self-reported measure of physical activity over the last 7 days [31], and (5) the 14-item Diabetes Knowledge Test [32] and the 5-item ABC test [33], both tests of diabetes-specific knowledge.

Participants then watched the narrated simulation on a laptop computer while wearing headphones. During the narrated simulation, all participants completed the two drawing tasks described above. To minimize demand effects, the investigator left the room while participants watched the animation; most questionnaires were administered by paper. However, since the IPAQ was going to be readministered by phone a week later, this questionnaire was administered orally by the investigator during the in-person meeting.

After participants watched the narrated simulation, the 14-item theory of planned behavior-related questionnaire and both diabetes-related knowledge tests were repeated. In addition, to measure the degree to which participants felt that the information in the animation was personally relevant, participants answered two 7-point Likert-type questions: “I think the glucose curves in the movie were related to *my diabetes*” and “I think the complications shown in the movie *could happen to me*.”

To conclude the in-person meeting, we asked participants about their qualitative impressions of the narrated simulation: what they liked and did not like, if there were parts of the simulation they found confusing, and if there were concepts they would like to see presented in this manner that were not included in the narrated simulation. These questions were administered orally.

We contacted participants by phone 1 week later and readministered the IPAQ measure of physical activity over the last 7 days [31]. This was followed by a questionnaire asking whether the participant thought about the glucose curves in the week since watching the narrated simulation and, if so, whether they thought about them before, during, or after eating, exercising, or testing their glucose. The purpose behind these last questions was exploratory for future work with this intervention.

### Analysis

We performed all analyses using R version 2.10.0, freely available statistical computing software [34]. We excluded 2 individuals from this analysis: 1 control participant who reported walking 35 hours/week at baseline and 1 intervention participant who reported walking 18 hours/week; these individuals’ baseline walking times were ≥2.5 standard deviations above the mean. In addition, including these individuals would have overestimated the effect of the intervention in our main hypotheses.

To test our primary hypothesis (that the intervention version of the narrated simulation would more positively affect individuals’ walking), we used a linear model with intervention status and preanimation walking (minutes/week) as the covariates. We adjusted for significant between-group differences in age and a near-significant difference in baseline behavioral intent (see Table 2).

To test our second hypothesis (that among intervention participants change in outcome expectancies [beliefs] would be a function of the discrepancy between prior beliefs and those presented in the narrated simulation), we first needed to calculate the change in outcome expectancy and then calculate a score reflecting the discrepancy between the presented outcome and the individual’s expected outcome. Once these scores were calculated, we used a linear model with an interaction between the discrepancy score and intervention status as a covariate after adjusting for age and baseline intent.

Outcome expectancies were measured using the following questions on the theory of planned behavior questionnaire: “Walking for at least 30 minutes will lower my blood sugar,” and “Walking for at least 30 minutes/day, 5 days a week *over the next 3 months *will lower my hemoglobin A1c.” Participants agreed or disagreed on a 7-point Likert scale (see Multimedia Appendix 3). As suggested by Azjen, for each of the pre- and post-theory of planned behavior measures, the individual’s score for these two questions was averaged to reflect the overall construct of outcome expectancy [30]. A change score was calculated by subtracting the preintervention measure of outcome expectancy from the postintervention measure.

**Figure 4:**
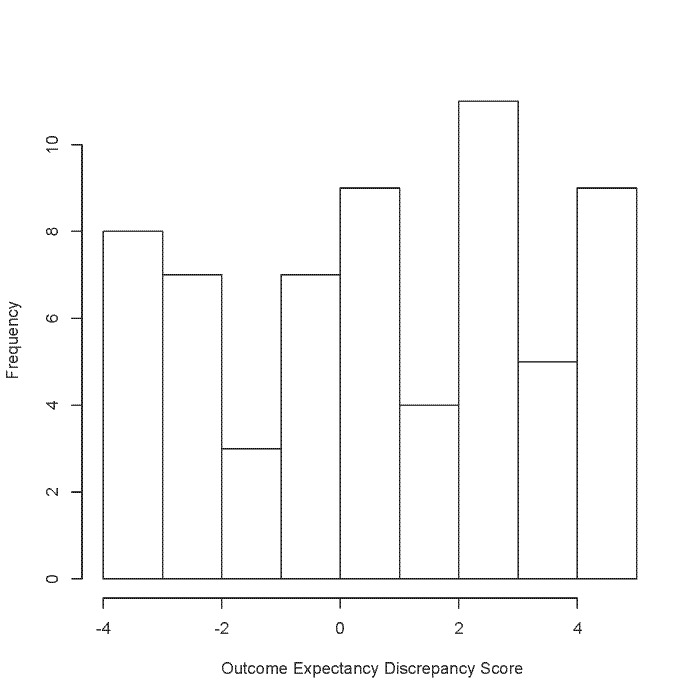
Distribution of outcome expectancy discrepancy scores.

We calculated the outcome expectancy discrepancy score by measuring the difference between the presented change in the glucose curve and the individual’s outcome expectancy elicited in the drawing task. We scored each dimension of the individual’s outcome expectancy (direction, duration, and magnitude) according to whether the individual’s outcome expectation was negative, neutral, or positive. For example, if the decrease in the individual’s drawn curve was greater in magnitude than the decrease in the presented curve (positive expectancy), this dimension was scored 1. If the magnitude of the participant’s expectation was the same as the presented curve, the score was 0 (accurate understanding). If the drawn magnitude was less than the presented curve, the participant was scored –1 (negative expectancy). Since the direction of the change in the curve could only increase or decrease, individuals were scored 1 if their drawing reflected a decrease (a positive expectancy and accurate understanding) and –1 if their drawing reflected an increase in blood glucose postexercise (negative expectancy). The discrepancy score used in the regression is the sum of all the dimension scores for both drawing tasks with a possible range of –6 to 6. Figure 4 is a histogram of the distribution of discrepancy scores.

To test our secondary hypotheses (that, overall, both versions of the narrated simulation would positively affect behavioral intentions and knowledge), we used paired *t *tests to compare presimulation versus postsimulation measures.

Finally, we conducted an exploratory analysis to inform future work by examining participants’ responses to the qualitative questions of what they liked and did not like in the narrated simulation, what they found confusing, and what they would like to see in future versions for recurrent themes. We also examined the proportion of individuals who reported thinking about the glucose curves in the next week and the context in which they reported thinking about them.

## Results

### Description of the Sample

Table 2 presents the baseline characteristics of the intervention and control groups. The randomization resulted in equal groups on all measures with the exception of age; the average age of the control group was slightly higher than that of the intervention group; in addition, a near-significant difference existed in baseline intentions regarding walking in the intervention group.

**Table 2:** Baseline characteristics of control and intervention groups.

Characteristic		Intervention group (n = 33)	Control group (n = 32)	*P *value
**Sex, n** ^a^				.87
	Male	20	21	
	Female	13	11	
Veterans, n^a^		10	12	.72
Age (years), median (range)^b^		56 (34–70)	61 (36–70)	.02
Years since diagnosis, median (range)^b^		7 (.02–20)	8.5 (.12–19)	.96
Hemoglobin A1c, median (range)^b^		7.0 (5.6–11.8)	6.9 (6.1–10.3)	.63
Diabetes numeracy (scale of 0–10), median (range)^b^		8 (1–10)	8 (2–10)	.34
Frequency of self-monitoring (times/week), median (range)^b^		5 (0.1–21)	2.75 (0–21)	.13
Have email?, n^a^		29	29	.96
Frequency of non-job email use (x/week), median (range)^b^		14 (0–14)	14 (0–14)	.65
Have a personal health record?, n^a^		12	10	.86
Nonwalking physical activity (metabolic equivalents × minutes/week), median (range)^b^		960 (0–8820)	512 (0–8640)	.12
Walking (minutes/week), median (range)^b^		90 (0–1080)	145(0–2100)	.27
Knowledge (Diabetes Knowledge Test, scale of 0–14), median (range)^b^		12 (5–14)	12 (6–14)	.55
Behavioral intention (scale of 1–7), median (range)^b^		5 (1–7)	6 (1–7)	.08

^a ^Chi-square test.

^b ^Kruskal-Wallis test.

### Hypothesis 1

**Figure 5:**
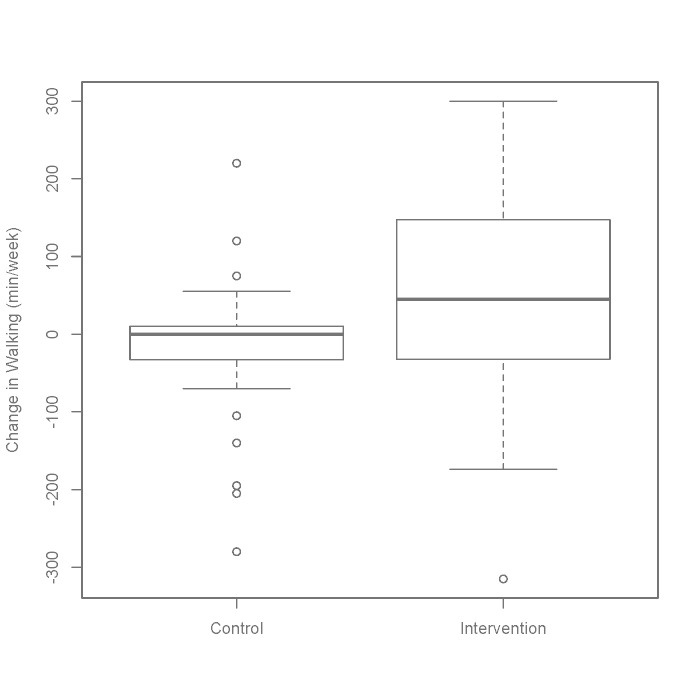
Change in walking by condition. Box: 1st-3rd quartile, whiskers: 1.5*interquartile range, circles: outliers.

Our first and most clinically significant hypothesis was supported: intervention participants increased walking time more than control participants. After taking into account baseline walking and adjusting for age and baseline behavioral intent, the mean effect of the intervention was an increase of 61.0 minutes (SE 30.5, *t*_58 = 1.9,_ *P *= .05). Neither age (coef = –1.2, SE 1.9, *t*_58 = –0.6,_ *P *= .5) nor baseline behavior intent (coef = 3.6, SE 9.5, *t*_58 = 0.3,_ *P *= .7) was a significant predictor of the change in walking. Figure 5 presents the change in walking by intervention status.

### Hypothesis 2

Our second hypothesis was supported: among intervention participants, the discrepancy between the individuals’ prior beliefs and the presented outcomes was associated with their change in outcome expectancy. The coefficient for the interaction between intervention status and discrepancy score was –.25 (SE .07, *t*_57 = –3.2,_ *P *< .01), indicating that on average, after viewing the simulation, the beliefs of individuals with negative baseline beliefs became more positive while the beliefs of those with overly optimistic baseline beliefs became more negative.

### Hypotheses 3 and 4

Our secondary hypotheses were also supported: both groups increased behavioral intentions, mean difference 0.66 on a scale of 7 (*t*_62 = 4.5,_ *P *< .001), and knowledge, mean difference 0.38 on a scale of 14 (*t*_62 = 2.4,_ *P *= .02). Table 3 summarizes hypotheses 1 and 2 and their results. Table 4 summarizes hypotheses 3 and 4 and their results. Table 5 presents the means and standard deviations for all outcome measures.

**Table 3:** Summary of hypotheses and results.

Hypothesis	Model	Coefficient	SE	*t *value	*df*	*P *value
Walking will increase more in intervention participants	Linear model regressing postintervention walking on intervention status, preintervention walking adjusted for age and preintervention intent	61.0	30.5	1.9	58	.05
Among intervention participants, change in outcome expectancy will be a function of the discrepancy between prior beliefs and the presented outcome	Linear model regressing the change in outcome expectancy on an interaction term between intervention status and discrepancy score, adjusted for age and preintervention intent	–.25	.07	–3.213	57	<.01

**Table 4:** Summary of hypotheses and results

Hypothesis	Model	Mean difference	*t *value	*df*	*P *value
Both group will increase in behavioral intention	Paired *t *test comparing postintervention versus preintervention measure	0.66	4.5	62	<.001
Both groups will increase in diabetes-related knowledge	Paired *t *test comparing postintervention versus preintervention measure	0.38	2.4	62	.02

**Table 5:** Means (SD) for all outcome measures pre- and postintervention.

Outcome measure	Intervention status	Pre intervention	Postintervention
Walking (minutes)	Intervention	182.9 (245)	230.3 (262)
	Control	203.5 (203)	185.6 (193)
Outcome expectancy (scale 1–7)	Intervention	6.07 (1.1)	6.56 (.82)
	Control	6.37 (.89)	6.69(.55)
Behavioral intent (scale 1–7)	Intervention	4.79 (1.62)	5.62 (1.80)
	Control	5.53 (1.60)	6.03 (1.24)
Knowledge (scale 1–14)	Intervention	11.15 (2.3)	11.71 (2.14)
	Control	11.29 (1.95)	11.48 (2.18)

### Qualitative Themes

We coded responses to qualitative questions into general themes and determined the proportion of each theme. When asked “What were the things that you liked about the simulation?” 31/65 of participants’ responses were coded as *informative*: these included comments such as “I thought the simulation was very clear” and “I think it was better than what I got in diabetes education.” Other themes that emerged were *surprise*: 11/65 participants commented that they were surprised at the effect of walking on the glucose curve. A third theme was *complications*: 7/65 participants reported liking the inclusion of pictures of individuals with complications; as 1 participant said, she felt that this was “important for people to see what might happen to them.” Finally, 5 participants reported that they had not seen or thought of glucose as a curve before, and 4 participants reported that they were previously unaware of the dawn phenomenon.

When asked “Were there things you did not like about the simulation?” most participants (52/65) answered “No.” Of those who provided specific negative feedback (13/65), 4 reported that the simulation contained “nothing new” or was “not interesting.” A total of 2 participants, both of whom worked nights and slept during the day, reported feeling that the content of the simulation was not relevant to them. In addition, 3 reported not liking the music or voiceover, 1 reported not liking the glucose curves, 1 reported not liking the drawing task, 1 reported not liking the numeracy test, and 1 thought the simulation was too slow in the beginning.

When asked “Were there parts of the simulation you found confusing or that brought up questions in your mind?” most participants (59/65) answered “No.” Of those who provided specific feedback, 3 reported finding the drawing task confusing and 2 reported not understanding the meaning of the curves.

When asked “Are there things that were not in the simulation that you would like to see in a simulation like this?” 9 participants commented they would like to see the effect of different foods on the glucose curve, 5 wanted more information about how the disease progresses over time and whether it is reversible, 4 commented that they would like to see numbers on the curves, 3 commented that they would like to see more answers to the test questions addressed in the narrated simulation (not all the questions on the knowledge tests were addressed in the simulation), 2 commented that they would like to see the effect of insulin, and 2 control participants wanted to see the effect of exercise on the curve.

Although there was a small difference in the proportion of individuals who reported thinking about the glucose curves in the week following the simulation by condition (27/33 intervention participants, 22/32 controls), this difference was not significant (χ^2^_1 = .88,_ *P *=. 35). When asked whether they thought about the glucose curves in the context of specific self-management behaviors, the proportions of all participants were as follows: when exercising (38/65), eating (35/65), and testing their blood sugar (30/65). There was no difference between groups in the incidence of thinking about the glucose curves in these contexts.

## Discussion

This study had two main findings. First, intervention participants who completed an action plan for walking in the next week reported significantly more walking in the subsequent week than control participants. This findings is congruent with a large number of both laboratory and clinical studies that have found a positive impact of implementation intentions and action plans [12]. Our use of an action plan with simultaneous mental simulation of the plan is not novel. However, prior studies used a healthy university student population [23,24]; this study used an older diabetic population.

Our second main finding was that intervention participants’ beliefs changed in accordance with the discrepancy between their prior beliefs and the outcomes presented in the simulation. The idea that computerized simulations could change outcome expectancies was suggested by Bandura in 1999 [35] and is in line with his earlier work demonstrating that individuals’ beliefs change as a result of their observations of the effects of their own and others’ behaviors [36]. We are unaware of any studies that have translated these ideas into a patient-facing intervention. We believe this finding suggests that computerized simulations could be used much more broadly to change individuals’ health-related beliefs.

We are aware of only one other study involving glucose curves to promote physical activity among individuals with T2DM. Allen et al randomly assigned 52 individuals to one-on-one educational sessions [37]. The intervention session incorporated glucose curves to demonstrate the effect of physical activity on glucose. The session also included discussing the benefits of increased activity, assessing the individual’s barriers to physical activity and self-efficacy for exercise, and providing an appropriate exercise prescription. The control session mentioned but did not stress physical activity as a self-management behavior for T2DM. At the 8-week follow-up, individuals in the intervention group had significantly greater improvements in self-efficacy for physical activity, accelerometer-measured physical activity, hemoglobin A1c, and body mass index. Both our study and Allen and colleagues’ used glucose curves to promote physical activity in individuals with T2DM, but there are important differences. First, the proposed mechanisms are different: the Allen intervention was intended to increase physical activity by increasing participants’ self-efficacy, while our intervention was intended to increase physical activity by changing outcome expectancies and implementing an action plan. Second, the degree of experimental control is different: our study was a comparison between two computerized simulations that differed only in the inclusion of two components; Allen and colleagues’ study compared in-person interventions that differed in many respects. We believe that these two studies, taken together, provide evidence that the outcome expectancies and self-efficacy of individuals with T2DM can be positively affected by modeling using glucose curves.

### Implications for Translation

The results of this study highlight the potential for the translation of specific evidence from the psychology literature into the design of informatics-based behavioral interventions. We used an action planning intervention to facilitate subsequent action in intervention participants. This technique holds great promise to facilitate health-related behaviors, particularly in mobile phone-based interventions. In fact, recent evidence has shown that sending text message reminders of planned actions further facilitates the desired action [38]. We also presented potential outcomes as upward counterfactuals (how things might have been better) to maximize participants’ behavioral intentions. This framing of information might be more widely used in consumer health informatics to increase user motivation; however, since we did not experimentally test this component of our intervention, further work is needed to test this idea.

### Strengths

This study has several strengths. First, we employed prior findings in the psychological literature to design a brief, self-contained intervention and conducted a hypothesis-driven test of the efficacy of components of the intervention. Second, our use of glucose curves for both the presentation and elicitation of outcomes allowed for the measurement of individuals’ outcome expectancies across three dimensions: the magnitude, duration, and direction of the effect. We believe this method is superior to the more common Likert scale measures of belief, and that a computer-based version of this drawing task could further improve upon the discrepancy score used in this study. A limitation of the discrepancy score used in this study is that it does not account for differences in the magnitude and duration of the individual’s expectation (a larger discrepancy reflects a more inaccurate belief than a smaller discrepancy). A better measure of the discrepancy would be the difference in the area under the curve between the individual’s curve and the presented outcome. This was not feasible using the complex curves drawn on paper in this study, but a computer-based version of the drawing task could easily calculate this difference.

### Limitations

This study has limitations. First, our primary outcome measure, physical activity, was measured by self-report. Since all participants used the same measure, we do not believe this undermines the results; however, the true magnitude of the effect of our intervention on subsequent physical activity needs to be determined with objective measures in future work. Additionally, some of our participants did not represent the target population for this intervention: some participants possessed adequate diabetes-related numeracy, had positive outcome expectancies and intentions for exercise, were knowledgeable about their disease, and were already physically active. We plan to address this issue in the future by integrating the intervention into diabetes education classes in target populations, particularly groups with newly diagnosed T2DM and low diabetes numeracy. The third limitation of this study was that the tests used to measure knowledge were not well aligned with the simulation’s presentation of content. We developed the simulation around gists we considered important based on theory [39], our clinical experience, and pilot work. Available measures of diabetes-related knowledge, including those used in this study, measure an individual’s knowledge of facts. Instruments measuring conceptual understanding of diabetes self-management are not available. In future work, simple simulations such as those used in this study could serve as a method to both teach and test understanding of diabetes-related concepts. A final limitation of this study is that, while we attempted to minimize the interaction between the investigator and the participant, some interaction was necessary (eg, the administration of the IPAQ). Further work is needed to determine the effectiveness of an entirely computer-based version of the intervention.

### Future Research

The next generation of this intervention will test the effectiveness of personalizing the feedback provided in an interactive phone-based intervention. A phone-based intervention may facilitate integration of the simulation into the user’s daily life, may be easier to access than traditional diabetes education, which reaches a limited population [40], and might be less costly than an in-person intervention [41]. Recently, Fisher et al [42] and Polonsky et al [43] reported on an in-person intervention called the Structured Test Protocol. The core of this intervention was the estimation of the individual’s glucose curves using 7-point glucose monitoring for 3 days. In their study, estimated curves facilitated shared decision making between patient and provider, resulting in a greater improvement in hemoglobin A1c, diabetes self-efficacy, autonomous motivation for diabetes care, and a more positive attitude toward self-monitoring of glucose than usual care [42]. This protocol concentrates the timing of self-monitoring but does not require a net increase in the volume of glucose monitoring [43], and therefore may be a cost-neutral and minimally invasive method to tailor the curves presented in the simulation. We hypothesize that the personalization of the presented curves, in combination with the personalization of the predicted effect of exercise (a subject of current research), may result in greater effectiveness of the intervention.

### Conclusion

In this study we tested a simple form of a computer-based simulation. Participants’ outcome expectancies changed in accordance with the discrepancy between their prior beliefs and the presented outcomes. In combination with action planning, the simulation positively affected short-term behavior.

## Multimedia Appendix 1

Intervention simulation.

## Multimedia Appendix 2

Control simulation.

## Multimedia Appendix 3

Theory of Planned Behavior Questionnaire.

## Abbreviations

IPAQ: International Physical Activity Questionnaire
T2DM: type 2 diabetes mellitus
