# Supplementary material for: Efficacy of a Computerized Simulation in Promoting Walking in Individuals With Diabetes
Source: J Med Internet Res. 2012 May 10;14(3):e71. doi: 10.2196/jmir.1965 (PMC3799542; doi:10.2196/jmir.1965)
Supplement: Supplementary file 3 [file jmir_v14i3e71_app3.pdf]

Please mark the line which shows how you think or feel.

1. For me to walk for at least 30 minutes/day for 5 days in the next week is:
unhealthy:____:_____:_____:_____:_____:_____:_____: healthy

2. My doctor thinks that
I should:___:_____:_____:_____:_____:_____:_____: I should not
walk for at least 30 minutes/day for 5 days in the next week

3. When it comes to walking, how much do you want to do what your doctor thinks you should do?
not at all :_____:_____:_____:_____:_____:_____:_____: very much

4. I intend to walk for at least 30 minutes/day for 5 days in the next week
extremely unlikely :_____:_____:_____:_____:_____:_____:_____: extremely likely

5. If I wanted to, I could walk at least 30 minutes/day for 5 days in the next week
definitely true :_____:_____:_____:_____:_____:_____:_____: definitely false

6. My family thinks that
I should:_____:_____:_____:_____:_____:_____:_____: I should not
walk for at least 30 minutes/day for 5 days in the next week

7. When it comes to walking, how much do you want to do what your family thinks you should do?
not at all :_____:_____:_____:_____:_____:_____:_____: very much

8. Walking for at least 30 minutes/day, 5 days a week *over the next 3 months* will lower my Hemoglobin A1c (average blood sugar).
extremely unlikely :_____:_____:_____:_____:_____:_____:_____: extremely likely

9. Lowering my blood sugar is
extremely bad :_____:_____:_____:_____:_____:_____:_____: extremely good

10. I will try to walk for at least 30 minutes/day for 5 days in the next week
definitely true :_____:_____:_____:_____:_____:_____:_____: definitely false

11. For me to walk at least 30 minutes/day for 5 days in the next week would be
impossible :_____:_____:_____:_____:_____:_____:_____: possible

12. Walking for at least 30 minutes will lower my blood sugar.
extremely unlikely :_____:_____:_____:_____:_____:_____:_____: extremely likely

13. Lowering my Hemoglobin A1c (average blood sugar) is
extremely bad :_____:_____:_____:_____:_____:_____:_____: extremely good

14. For me to walk for at least 30 minutes/day for 5 days in the next week is:
pleasant: _____:_____:_____:_____:_____:_____:_____: unpleasant
